# Triage implementation in resource-limited emergency departments: sharing tools and experience from the Pacific region

**DOI:** 10.1186/s12245-024-00583-8

**Published:** 2024-02-14

**Authors:** Rob Mitchell, Libby White, Leigh Elton, Cliff Luke, Sarah Bornstein, Vincent Atua

**Affiliations:** 1 School of Public Health & Preventive Medicine, Monash University, Melbourne, Australia; 2 Emergency & Trauma Centre, Alfred Hospital, Melbourne, Australia; 3 National Critical Care & Trauma Response Centre, Darwin, Australia; 4 Vila Central Hospital, Port Vila, Vanuatu; 5 Respond Global, Noosa, Australia

## Abstract

According to the World Health Organization’s (WHO) Emergency Care Systems Framework, triage is an essential function of emergency departments (EDs). This *practice innovation* article describes four strategies that have been used to support implementation of the WHO-endorsed Interagency Integrated Triage Tool (IITT) in the Pacific region, namely needs assessment, digital learning, public communications and electronic data management.

Using a case study from Vila Central Hospital in Vanuatu, a Pacific Small Island Developing State, we reflect on lessons learned from IITT implementation in a resource-limited ED. In particular, we describe the value of a bespoke needs assessment tool for documenting triage and patient flow requirements; the challenges and opportunities presented by digital learning; the benefits of locally designed, public-facing communications materials; and the feasibility and impact of a low-cost electronic data registry system.

Our experience of using these tools in Vanuatu and across the Pacific region will be of interest to other resource-limited EDs seeking to improve their triage practice and performance. Although the resources and strategies presented in this article are focussed on the IITT, the principles are equally relevant to other triage systems.

**Supplementary Information:**

The online version contains supplementary material available at 10.1186/s12245-024-00583-8.

The World Health Organization’s Emergency Care Systems Framework summarises the critical components of high-quality emergency care systems [1]. According to this guidance, one of the essential functions of emergency departments (EDs) is triage. This is broadly defined as the process of sorting patients according to the urgency of their presentation, such that those with time-critical care needs are prioritised for assessment and management [2, 3].

This *practice innovation* article describes four strategies that have been utilised to support triage implementation and quality improvement in the Pacific region, namely needs assessment, digital learning, public communications and electronic registries. In sharing our experience and resources, we hope to enable triage introduction and performance enhancement in low- and middle-income country (LMIC) EDs.

## Background

A small number of triage tools have been specifically developed for resource-constrained emergency care settings [4, 5]. This includes the Interagency Integrated Triage Tool (IITT), a three-tier, colour-coded triage system endorsed by the World Health Organization, Médecins Sans Frontières and International Committee of the Red Cross [6–8]. The IITT has been designed to enable a simple and efficient approach to triage, cognisant of the challenges for triage practice where human and other resources are limited [9].

Although the IITT has been implemented in a number of LMICs, including Bangladesh, Honduras and Somalia [8, 10, 11], most (if not all) data on the tool’s performance emanates from the Pacific region [12–15]. This reflects that triage improvement is a priority for Pacific Island Countries and Territories (PICTs) [16, 17], and the initial IITT validation studies were undertaken in Papua New Guinea (PNG) [12, 13]. Given the tool’s recent release (in early 2020) [6], facilities in other regions may not yet have had the opportunity to publish their experience and evaluation findings.

The IITT is now in operation in EDs across a number of PICTs, including PNG, Kiribati and Vanuatu. Implementation efforts have been heavily informed by early pilot studies in Port Moresby and Mount Hagen, [7, 12, 13] and experience with the Solomon Islands Triage Scale (a similar, three-tier triage tool) in Honiara [18]. These quality improvement projects emphasised the importance of rigorous approaches to needs assessment, training, public communications and performance monitoring [7, 12, 13, 18].

In this article, we summarise the rationale, and an approach, for each of these strategies. By way of a case study, the accompanying boxes describe lessons learned from Vila Central Hospital (VCH), Vanuatu’s national referral hospital.

As with many PICTs, Vanuatu is a Small Island Developing State [19]. Challenges in the delivery of healthcare include a geographically dispersed population and an escalating burden of noncommunicable disease [19–22]. Although Vanuatu’s emergency care system is under-developed, the value of timely and quality acute care has been recognised by the Ministry of Health [20–22].

Prior to the onset of this project, there was no consistent, systematised approach to triage at VCH. The objective of this initiative was to implement the IITT, and then establish a process for quality monitoring and ongoing improvement. This work was instigated by the leadership team at VCH ED in response to local concerns about delays to care for urgent patients, primarily due to overwhelming demands from non-acute presentations and the absence of a clear system for identifying those with time-sensitive care needs.

Improving triage quality at VCH was undertaken as a collaborative emergency care development project between ni-Vanuatu and Australian clinicians. The principles of this type of approach have been described previously but include co-design and long-term partnership [7, 23, 24]. Support from Australian emergency nursing advisors deployed to VCH through the Australian Volunteers Program, an Australian Government initiative, was critical to this work.

## Practice innovation

### Needs assessment

A comprehensive understanding of local needs and context is critical to the success of any global emergency care development project. The role of broad-based ED needs assessments, and the tools available to facilitate them, have been described in detail elsewhere [25].

To our knowledge, there is no published needs assessment instrument specifically focussed on triage implementation. For the purpose of this project, a bespoke ‘Emergency Department Systems Assessment Tool’ (EDSAT) was developed. The objective of this proforma was to capture all necessary information relevant to triage, patient flow and data management in the ED, with a view to identifying priority areas for improvement.

The tool was structured around previously described building blocks for Pacific emergency care: human resources, infrastructure and equipment, data, processes, and leadership and governance [16]. An additional section, focussed on case mix, was also included.

The EDSAT proforma, as deployed at VCH, is available at Appendix A. This version of the tool was developed in a pre-pandemic context, so does not include content relevant to the screening and care of patients with suspected or confirmed COVID-19. The template could easily be adapted, however, to incorporate further information related to infection prevention and control.

The EDSAT has subsequently been deployed across several EDs in PICTs and Timor-Leste. It has enabled triage implementation strategies to be targeted to the specific needs and characteristics of the ED. For example, it has identified where clinical redesign and workforce strengthening have been required (Text box 1).

**Text box 1:** Needs assessment — reflections and lessons learned from VCH

Prior to the initiation of this project, improving triage and referral processes had been identified as a priority for the Vanuatu Ministry of Health and VCH [20, 22]. To ensure that all stakeholders had a shared understanding of the contemporary model of care and case mix in the ED, local and visiting clinicians collaboratively completed the EDSAT.
The EDSAT was administered by a visiting nurse advisor and local ED clinicians. Completing the proforma allowed the group to identify that, alongside triage training, a number of other systems improvements would be required. For instance, the process resulted in reallocation of the clinical space dedicated to resuscitation, to better align with the IITT’s patient flow principles. It also highlighted the potential gains that would come from integration of the existing outpatient department with the ED, to ensure a consistent model of care at the ‘front door’ of the hospital.
Completing the EDSAT emphasised the need for a holistic and multidisciplinary approach to the patient care journey. It affirmed the importance of including the local ambulance service in training and systems changes, and the need for positive engagement with inpatient wards to support effective patient flow into and out of the ED.
Finally, the EDSAT identified the need to optimise nursing staffing to ensure that a nurse could be allocated to the triage role for each shift, as well as each of the care streams implemented as part of the new model (resuscitation, acute and fast track). It also reinforced the need for local ‘champions’ to lead the quality improvement process. Although workforce reform was beyond the scope of the triage project, the EDSAT highlighted the interconnectedness of different emergency care building blocks (i.e., that improving ‘processes’ is also dependent on ‘human resources’ and ‘leadership and governance’.).

Completion of the EDSAT has provided an opportunity for leaders to collectively discuss their concerns and priorities before the commencement of any interventions. In our experience, this has helped foster a collaborative and multidisciplinary approach from the outset.

Additionally, the tool provides a valuable means of documenting contemporary practices and policies, which then become a historical reference point. This enables progress to be tracked over time. Lessons learned from VCH in relation to the EDSAT are provided in Text box 1.

### Digital learning

There is broad recognition that digital learning is a useful tool for enhancing knowledge and capability among healthcare workers in LMICs [26, 27]. However, there is relatively little data on the feasibility and effectiveness of online education for improving emergency care practice, especially in resource-limited settings where Internet access can be challenging [26–28].

The COVID-19 pandemic mandated innovative approaches to global emergency care collaboration, development and knowledge translation [29]. This stimulated the development of novel digital learning tools, including the roll-out of platforms and materials specifically focussed on triage. For example, Médecins Sans Frontières’ Tembo system features a dedicated module on the IITT [30]. Although the efficacy of this particular learning package has not been assessed, a recent evaluation of the broader Tembo platform found it to be a functional tool for meeting the training needs of clinicians and other field workers [31].

In PNG, a bespoke learning management system was developed to facilitate implementation of the IITT at two large referral hospitals [32]. All imagery utilised on the platform was purposely designed to ‘look and feel’ Melanesian, and content was delivered using micro-learning principles. This concept is well suited to low-resource settings, where smartphones are often used to access online learning and bandwidth can be limited. A comprehensive evaluation of this programme has been published elsewhere, verifying the value of digital learning in the Pacific context [28].

**Fig. 1:**
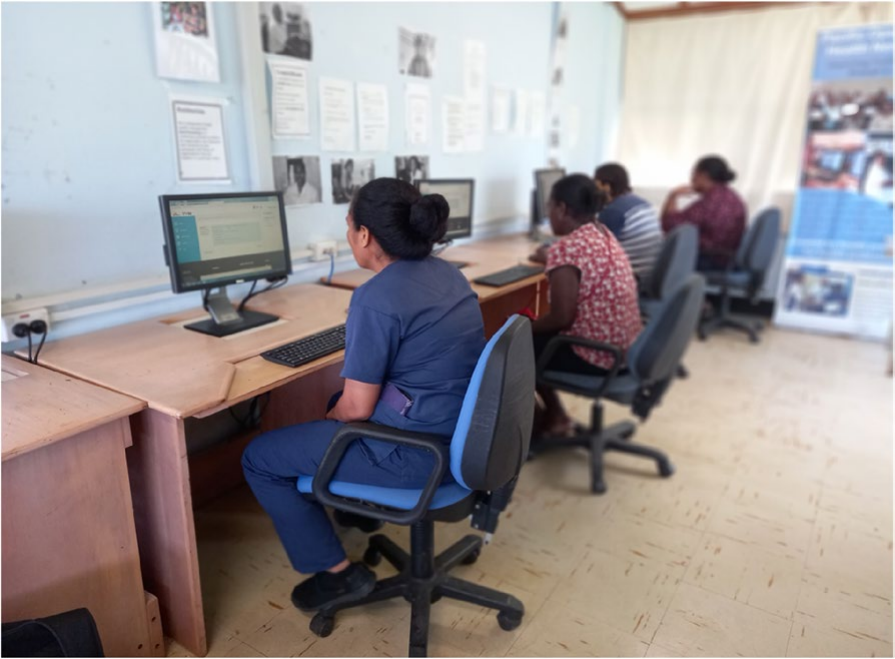
VCH staff completing IITT digital training in the hospital’s computer laboratory

Data from PNG, and elsewhere, supports blended approaches to triage and emergency care training, where digital learning is supported by peer mentoring and in-person tuition, particularly for procedural skills [28, 33]. This approach balances efficiency and effectiveness, ensuring that cost-effective, online learning strategies are supplemented by contextualised, face-to-face content delivery. Reflections on digital learning at VCH are summarised in Text box 2 and Fig. 1.

**Text box 2:** Digital learning — reflections and lessons learned from VCH

The ED leadership team at VCH elected to support triage implementation with online learning for staff. The IITT module on Médecins Sans Frontières’ Tembo system was utilised [30].
As part of a multimodal approach to IITT training, clinicians were supported to access Tembo by visiting nurse advisors. Given that many VCH staff had never undertaken online learning, a hospital computer laboratory was utilised to ensure all participants were able to navigate the online modules (Fig. 1). When clinicians felt confident using the platform, they were able to continue their engagement with Tembo using computers in the ED.
Through an interval evaluation process, staff reported that use of the digital platform effectively augmented their learning. They found the modules to be highly beneficial because they were interactive and used case examples that were relevant to the VCH context. The prospect of a certificate of completion provided additional motivation.
The main challenge with this approach was computer literacy. As a manifestation of this, many clinicians did not have an email address, which is a Tembo login requirement. Visiting nurse advisors supported the creation of email addresses and encouraged local clinicians to set up an inbox on their smartphones for continued use. Although all nurses at VCH are issued with government email addresses, many had never accessed their accounts.
Despite these challenges, Tembo proved an effective means of conveying important triage principles and concepts. Additionally, because it is free to access, the platform remains an efficient and effective way of onboarding new ED staff.

### Public communications

Triage represents the first point of clinician contact for patients presenting to hospital. It is critical, therefore, that the public have an awareness of reception processes. This is particularly important in communities and cultures where certain population groups have traditionally been given priority access to healthcare. The concept of urgency-based triage can be challenging to communicate and implement in these contexts [34].

**Fig. 2:**
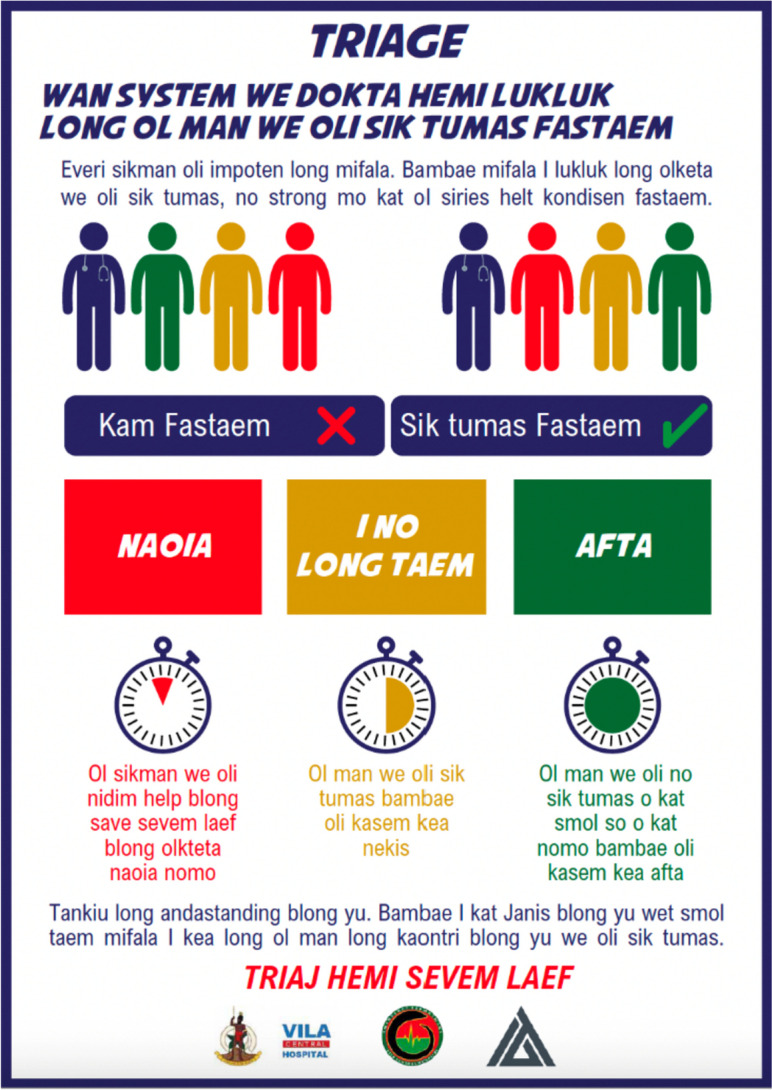
Explanatory poster in Bislama

In the Pacific, a variety of approaches to public education have been utilised. In the Western Highlands of PNG, for instance, public messaging via radio, television and local newspapers helped ensure community support for urgency-based triage [7, 13, 35–37]. In Vanuatu, as explained in Text box 3, a number of explanatory materials in local language were produced Fig. 2.

**Text box 3:** Public communications — reflections and lessons learned from VCH

Clinicians at VCH identified strategies that would be required for public education about triage. These included discussing the changes on the local radio and television station, as well as direct conversations with relevant stakeholders.
Clinicians considered creating a video demonstrating the triage process, to be displayed in the ED, however, the costs involved were prohibitive. Rather, a series of poster infographics in local language were developed that aimed to communicate triage principles (Fig. 2).
A local designer was commissioned to produce three different versions of the poster. The brief was to provide a visual representation of the triage process, using graphics to support the text.
Posters were initially produced in English. They were then reviewed and approved by the Ministry of Health and translated into Bislama and French (both national languages). The multilingual approach was used to ensure all community members could easily interpret the information.
The posters are now displayed in VCH’s reception area. They appear in a prominent position, so that all patients presenting for care can rapidly digest the information.

### Electronic data management

A systematic approach to data collection and reporting is essential to ED quality improvement [38, 39]. Notwithstanding the challenges associated with resource limitations, registry systems are widely recognised as an efficient and effective tool for collecting clinical and administrative data in LMIC emergency care settings [40].

With respect to triage, regular review of ED performance is recommended to monitor adherence to pre-specified time targets (for example the proportion of category 1 presentations seen within 5 minutes of arrival) and ensure that the selected triage tool remains practicable and valid in the particular context (namely, that it can efficiently and accurately identify patients with time-critical care needs) [2, 38, 41]. Although periodic audits can be utilised to collect this data, systematised collection of relevant indicators enables performance tracking over time [38, 40].

**Fig. 3:**
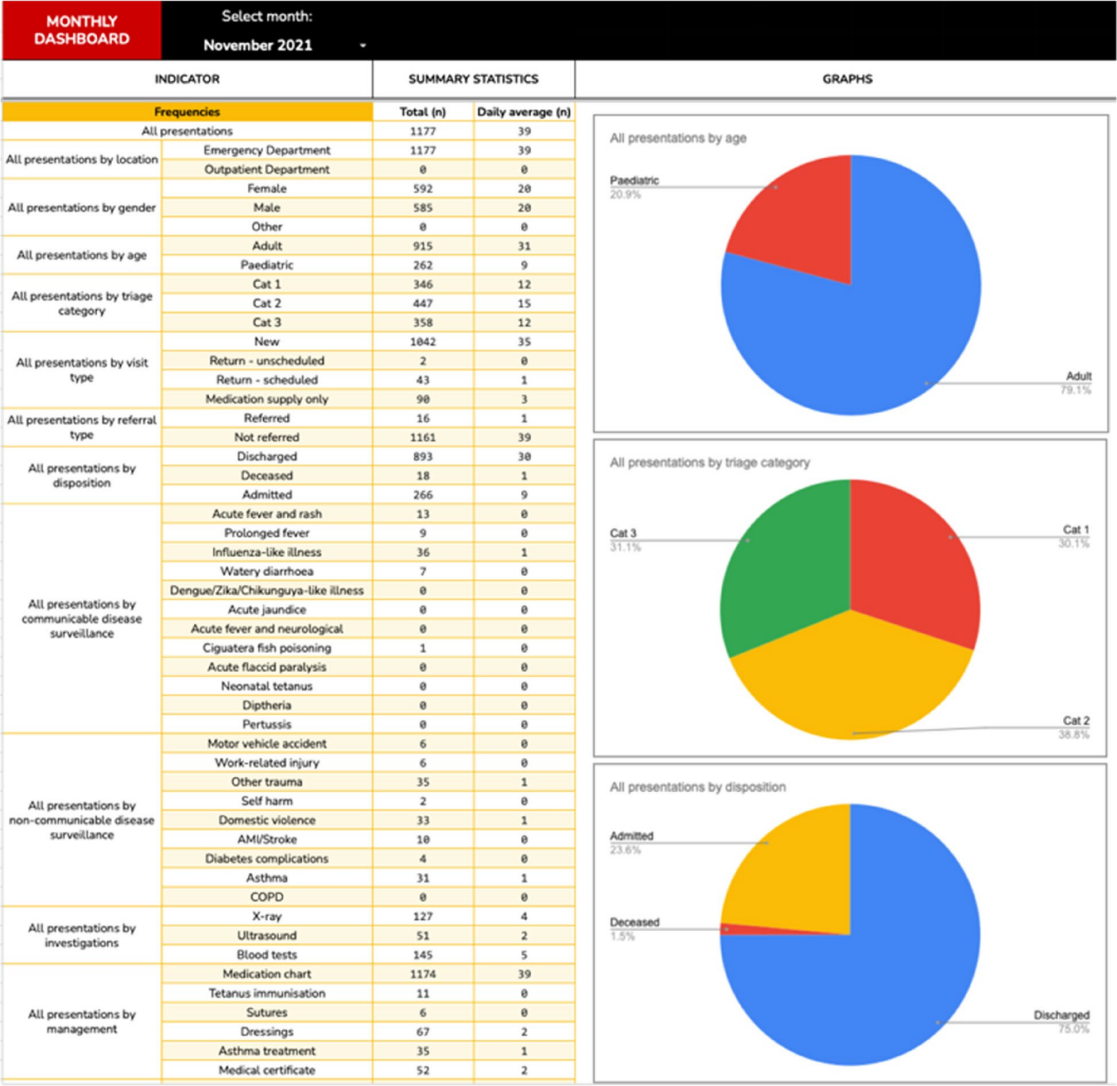
Screenshot of VCH’s electronic ED registry

In the Pacific, EDs in Solomon Islands and PNG have effectively implemented simple registry systems to enable collection and reporting of triage data [7, 18]. VCH’s experience in using this approach is summarised in Text box 4 and Fig. 3.

**Text box 4:** Electronic data management — reflections and lessons learned from VCH

Although VCH employs an electronic hospital information system (HIS), at the time this project was initiated, the system had not been implemented in the ED because a specific emergency care module was yet to be developed. While VCH staff considered utilising the interface designed for the outpatient department, it did not have sufficient functionality to capture and aggregate ED performance data.
An interim solution was developed to facilitate triage and emergency care data management while awaiting extension of the HIS to the ED. Step 1 involved reviewing the existing patient registration form and updating this to incorporate triage information and other relevant variables. This process was led by VCH staff and ultimately resulted in the rollout of a new clinical form that was better suited to local purposes.
Second, a simple electronic data entry form was developed to allow a data clerk to transfer handwritten information, in deidentified form, from the clinical form into an electronic database. Jotform (San Francisco, CA, USA) was used, because this platform is compliant with Health Insurance Portability and Accountability Act requirements (ensuring high standards of data protection) and allows offline access via its accompanying app. This functionality was thought to be important, because in the event of the loss of an Internet connection, data entry could be maintained. Data entered during offline periods is subsequently uploaded when a connection is re-established.
Third, a spreadsheet was designed within Google Sheets (Mountain View, CA, USA) to allow data aggregation and visualisation. Deidentified data entered into Jotform is automatically imported into this file through an integration function. This data is collated in a hidden worksheet, which functions as the data source for the rest of the spreadsheet. The entire document is password protected and requires two-factor authentication to access.
A series of worksheets were developed to function as dashboards. Formulas were used to calculate frequencies and percentages and present this data as tables and graphs for a variety of time periods (daily, weekly, monthly or yearly). For instance, a dashboard developed to aggregate monthly performance data displays data about the number of presentations, patient demographics and referral patterns, emergency care time intervals (e.g. time to assessment and total ED length of stay), compliance with time targets and disease surveillance. A screenshot of the frequency data is provided at Fig. 3.
A separate dashboard was created to facilitate the reporting of weekly syndromic surveillance data, as required by the Ministry of Health. This displays the presentation frequencies for 12 specific, pre-defined syndromes and diagnoses, identified by clinicians and recorded on the patient’s form at the time of the encounter. This data can then be submitted to the Ministry of Health as required.
The major impediment to the effective utilisation of this system has been data entry capacity. Although it requires less than 1 min to enter data from a patient’s clinical form into the electronic registry, remaining up to date with data entry has proved to be a challenge. This experience is not unique to VCH.
This simple, low-cost electronic registry has facilitated the capture and collation of important ED data. It has been used for reporting and audit purposes and has the potential to facilitate epidemiological and systems research. Overall, it has been a positive addition to the quality improvement toolkit at VCH ED.
On the back of this experience, VCH is now a pilot site for the WHO International Registry on Trauma and Emergency Care [42]. This new system uses the same workflows but a different registry platform, allowing comparison of data between sites and countries. An evaluation of the registry is expected to be published shortly.

The potential impact of systematised approaches to data collection cannot be overstated. Although this article is focussed on the value of data to the monitoring of triage performance, data registries have the potential to enhance a range of emergency care functions, including surveillance and research [40]. For this reason, the WHO is strongly encouraging uptake of its International Registry of Trauma and Emergency Care [42]. The experience from the Pacific, including VCH, has verified that the capture and reporting of quality data is feasible and effective when supported by adequate human resources and infrastructure.

Triage quality improvement also involves audit and review of triage officer performance, particularly in relation to adherence to assessment criteria. Some PICTs have developed processes for this, for instance mandating that every clinician who performs triage periodically undergo a period of direct supervision by a senior nurse. Practically, this involves each triage officer being actively observed by their senior colleague while undertaking triage assessments, providing an opportunity for feedback, continual improvement and credentialing.

## Discussion

Although triage is broadly recognised as an important function of emergency care systems, only a minority of PICTs are consistently and reliably using recognised triage tools [16]. In this article, we have summarised four strategies employed to support triage implementation and quality improvement across the region, with a focus on the experience at VCH in Vanuatu.

The COVID-19 pandemic has highlighted the importance of timely and quality emergency care [8, 43]. At the same time, demands on LMIC EDs have intensified, and there is a critical shortage of healthcare workers globally. This confluence of events has mandated new approaches to emergency care development and delivery, with a focus on strategies that enable optimal outcomes with efficient use of resources [17, 43]. ED triage meets these criteria.

Traditionally, the approach to implementing triage in LMIC settings has relied predominantly on in-person training of clinicians. The literature includes many examples of this strategy, across a variety of countries and contexts [18, 44–48]. Conversely, there are few published examples of alternate approaches.

Although the strategies detailed here are not unique, they demonstrate the value of a structured approach to triage introduction that leverages global experience in implementation science [49]. Specifically, the experience at VCH has reinforced the importance of a structured needs assessment that highlights potential barriers to effective triage, the importance of context-specific public communications, and a mechanism for data collection.

Implementation mentorship brings these strategies together [50]. While digital learning approaches have shown to be effective, the inclusion of mentoring (in-person and/or remote) is important to ensure process changes are sustainable and are adapted to the context. With respect to triage, the provision of sustained mentoring arrangements between local and external clinicians has supported implementation by providing continued momentum for change, and ongoing refinement of processes to align with operational needs. It has also facilitated a “complexity-informed” approach to systems improvement, by enabling frequent engagement with, and feedback from, those staff who are most affected by the reform [51].

Unsurprisingly, several barriers were encountered throughout this process. A fundamental and cross-cutting issue was workforce shortage, impacting the availability of clinical and administrative staff to complete triage training, and the ability to fill the triage officer role on an ongoing basis. This reflects that human resource capacity is a building block for emergency care systems, and a challenge across the Pacific region [16, 52, 53]. Similar issues have been reported in other settings [9, 41, 54].

We hope the experience reported here demonstrates the value of context-specific approaches to triage implementation. Clinicians in other LMICs are encouraged to utilise the highlighted resources to enhance the quality of triage in their departments. Given the potential of triage to reduce mortality and enhance ED functioning [55], this package of interventions may serve to improve outcomes in a wide variety of resource-limited settings.

### Supplementary Information

**Additional file 1:** **Appendix A.** Emergency Department Systems Assessment Tool.

## Data Availability

Not applicable.

## References

[1] World Health Organization. Emergency care systems framework. https://www.who.int/publications/i/item/who-emergency-care-system-framework. Accessed 28 Mar 2023.

[2] FitzGerald G, Jelinek GA, Scott D, Gerdtz MF (2010). Emergency department triage revisited. Emerg Med J.

[3] Mitchell R (2023). Triage for resource-limited emergency care: why it matters. Emerg Crit Care Med.

[4] Jenson A, Hansoti B, Rothman R, de Ramirez SS, Lobner K, Wallis L (2018). Reliability and validity of emergency department triage tools in low- and middle-income countries. Eur J Emerg Med.

[5] Hansoti B, Jenson A, Keefe D (2017). Reliability and validity of pediatric triage tools evaluated in low resource settings: a systematic review. BMC Pediatr.

[6] World Health Organization. Clinical care of severe acute respiratory infections – tool kit. https://www.who.int/publications/i/item/clinical-care-of-severe-acute-respiratory-infections-tool-kit. Accessed 1 Oct 2020.

[7] Mitchell R, McKup JJ, Bue O (2020). Implementation of a novel three-tier triage tool in Papua New Guinea: a model for resource-limited emergency departments. Lancet Reg Heal - West Pacific.

[8] Eaton L (2020). Emergency care in the pandemic. Bull World Health Organ.

[9] Ibrahim BE (2022). Sudanese emergency departments: a study to identify the barriers to a well-functioning triage. BMC Emerg Med.

[10] Argote-Aramendiz K, Jamieson J, Mitchell R (2022). A unique cup of coffee. When Minutes Matter.

[11] Sahsi N. Practicing EM in Bangladesh – build it and they will come. Emergency Medicine Cases. https://emergencymedicinecases.com/practicing-emergency-medicine-bangladesh/. Published 2022. Accessed 10 Jan 2023.

[12] Mitchell R, Bue O, Nou G (2021). Validation of the Interagency Integrated Triage Tool in a resource-limited, urban emergency department in Papua New Guinea: a pilot study. Lancet Reg Heal - West Pacific.

[13] Mitchell R, McKup JJ, Banks C (2022). Validity and reliability of the Interagency Integrated Triage Tool in a regional emergency department in Papua New Guinea. Emerg Med Australas.

[14] Mitchell R, Sebby W, Piamnok D (2023). Performance of the Interagency Integrated Triage Tool in a resource-constrained emergency department during the COVID-19 pandemic. Australas Emerg Care.

[15] Mitchell R, Kingston C, Tefatu R (2022). Emergency department triage and COVID-19: performance of the Interagency Integrated Triage Tool during a pandemic surge in Papua New Guinea. Emerg Med Australas.

[16] Phillips G, Creaton A, Airdhill-Enosa P (2020). Emergency care status, priorities and standards for the Pacific region: a multiphase survey and consensus process across 17 different Pacific Island Countries and Territories. Lancet Reg Heal - West Pacific.

[17] Mitchell R, O’Reilly G, Herron L (2022). Lessons from the frontline: the value of emergency care processes and data to pandemic responses across the Pacific region. Lancet Reg Heal - West Pacific.

[18] Wanefalea LE, Mitchell R, Sale T, Sanau E, Phillips GA (2019). Effective triage in the Pacific region: the development and implementation of the Solomon Islands Triage Scale. Emerg Med Australas.

[19] World Health Organization Western Pacific Regional Office (2017). Pacific Island Countries and Areas.

[20] Vanuatu Ministry of Health. National Referral Policy. Port Vila; 2019.

[21] Vanuatu Ministry of Health. Ministry of Health Workforce Development Plan 2019–2025. Port Vila; 2019.

[22] Atua V, Jamieson J, Mitchell R (2022). Brooms, mops and cookie jars. When Minutes Matter.

[23] Phillips GA, Hendrie J, Atua V, Manineng C (2012). Capacity building in emergency care: an example from Madang. Papua New Guinea Emerg Med Australas.

[24] Tassicker B, Tong T, Ribanti T, Gittus A, Griffiths B (2019). Emergency care in Kiribati: a combined medical and nursing model for development. Emerg Med Australas.

[25] Phillips G, Bowman K, Sale T, O’Reilly G (2020). A Pacific needs analysis model: a proposed methodology for assessing the needs of facility-based emergency care in the Pacific region. BMC Health Serv Res.

[26] World Health Organization. Digital Education for Building Health Workforce Capacity. Geneva; 2020.

[27] Craig A, Beek K, Godinho M (2022). Digital Health and Universal Health Coverage: Opportunities and Policy Considerations for Pacific Island Health Authorities.

[28] Mitchell R, Bornstein S, Piamnok D (2023). Multimodal learning for emergency department triage implementation: experiences from Papua New Guinea during the COVID-19 pandemic. Lancet Reg Heal - West Pacific.

[29] Karim N, Rybarczyk MM, Jacquet GA (2021). COVID‐19 pandemic prompts a paradigm shift in Global Emergency Medicine: multidirectional education and remote collaboration. Coates WC, ed. AEM Educ Train.

[30] Medecins Sans Frontieres. Tembo. https://tembo.msf.org. Accessed 23 Aug 2022.

[31] O’Neil G, Grosso S, Juillerat H, O’Neil T (2021). Tembo: the new learning and development platform.

[32] Tetang E. Implementing mobile learning in a Papua New Guinea hospital. https://catalpa.io/blog/mobile-learning-png-hospitals/. Accessed 6 Aug 2023.

[33] Savage AJ, McNamara PW, Moncrieff TW, O’Reilly GM (2022). Review article: e-learning in emergency medicine: a systematic review. Emerg Med Australas.

[34] Hassankhani H, Soheili A, Vahdati SS, Amin Mozaffari F, Wolf LA, Wiseman T (2019). “Me first, others later” a focused ethnography of ongoing cultural features of waiting in an Iranian emergency department. Int Emerg Nurs.

[35] Peki R (2019). Mt Hagen Hospital Pioneers New System. Post-Courier.

[36] EMTV. New emergency care system trialed at Mt Hagen Hospital. https://emtv.com.pg/new-emergency-care-system-trialed-at-mt-hagen-general-hospital/. Accessed 1 Nov 2019.

[37] Western Highlands Provincial Health Authority. New emergency care system for Mt Hagen Hospital. https://www.whhs.gov.pg/2019/05/new-emergency-care-system-for-mt-hagen-hospital/. Accessed 1 Sept 2019.

[38] Broccoli MC, Moresky R, Dixon J (2018). Defining quality indicators for emergency care delivery: findings of an expert consensus process by emergency care practitioners in Africa. BMJ Glob Heal.

[39] Hansen K, Boyle A, Holroyd B (2020). Updated framework on quality and safety in emergency medicine. Emerg Med J.

[40] Mowafi H, Ngaruiya C, O’Reilly G (2019). Emergency care surveillance and emergency care registries in low-income and middle-income countries: conceptual challenges and future directions for research. BMJ Glob Heal.

[41] King C, Dube A, Zadutsa B (2021). Paediatric emergency triage, assessment and treatment (ETAT) – preparedness for implementation at primary care facilities in Malawi. Glob Health Action.

[42] World Health Organization. WHO International Registry for Trauma and Emergency Care. https://www.who.int/publications/m/item/who-international-registry-for-trauma-and-emergency-care. Accessed 23 Jul 2023.

[43] Herron L-M, Phillips G, Brolan CE (2022). “When all else fails you have to come to the emergency department”: overarching lessons about emergency care resilience from frontline clinicians in Pacific Island countries and territories during the COVID-19 pandemic. Lancet Reg Heal - West Pacific.

[44] Molyneux E, Ahmad S, Robertson A (2006). Improved triage and emergency care for children reduces inpatient mortality in a resource-constrained setting. Bull World Health Organ.

[45] Robison JA, Ahmad ZP, Nosek CA (2012). Decreased pediatric hospital mortality after an intervention to improve emergency care in Lilongwe. Malawi Pediatrics.

[46] Duke T (2016). New WHO guidelines on emergency triage assessment and treatment. Lancet.

[47] Wangara AA, Hunold KM, Leeper S (2019). Implementation and performance of the South African Triage Scale at Kenyatta National Hospital in Nairobi, Kenya. Int J Emerg Med.

[48] Hategeka C, Mwai L, Tuyisenge L (2017). Implementing the emergency triage, assessment and treatment plus admission care (ETAT+) clinical practice guidelines to improve quality of hospital care in Rwandan district hospitals: healthcare workers’ perspectives on relevance and challenges. BMC Health Serv Res.

[49] Austin EE, Blakely B, Tufanaru C, Selwood A, Braithwaite J, Clay-Williams R (2020). Strategies to measure and improve emergency department performance: a scoping review. Scand J Trauma Resusc Emerg Med.

[50] Phillips G, Lee D, Shailin S, O’Reilly G, Cameron P (2019). The Pacific Emergency Medicine Mentoring Program: a model for medical mentoring in the Pacific region. Emerg Med Australas.

[51] Braithwaite J, Churruca K, Long JC, Ellis LA, Herkes J (2018). When complexity science meets implementation science: a theoretical and empirical analysis of systems change. BMC Med.

[52] Yamamoto TS, Sunguya BF, Shiao LW, Amiya RM, Saw YM, Jimba M (2012). Migration of health workers in the Pacific Islands. Asia Pacific J Public Heal.

[53] Brolan CE, Körver S, Phillips G (2022). Lessons from the frontline: the COVID-19 pandemic emergency care experience from a human resource perspective in the Pacific region. Lancet Reg Heal - West Pacific.

[54] Joshi N, Wadhwani R, Nagpal J, Bhartia S (2020). Implementing a triage tool to improve appropriateness of care for children coming to the emergency department in a small hospital in India. BMJ Open Qual.

[55] Mitchell R, Fang W, Tee QW (2023). Systematic review: what is the impact of triage implementation on clinical outcomes and process measures in low- and middle-income country emergency departments?. Acad Emerg Med.

